# Palliative care needs-assessment and measurement tools used in patients with heart failure: a systematic mixed-studies review with narrative synthesis

**DOI:** 10.1007/s10741-020-10011-7

**Published:** 2020-08-03

**Authors:** Bader Nael Remawi, Amy Gadoud, Iain Malcolm James Murphy, Nancy Preston

**Affiliations:** 1 Lancaster Medical School, Faculty of Health and Medicine, Lancaster University, Lancaster, LA1 4YG UK; 2 International Observatory on End of Life Care, Faculty of Health and Medicine, Lancaster University, Lancaster, LA1 4YG UK; 3 Trinity Hospice and Palliative Care Services, Low Moor Road, Blackpool, FY2 0BG UK

**Keywords:** Palliative care, Heart failure, Needs-assessment, Needs-measurement, Systematic review, Mixed-studies review, Narrative synthesis

## Abstract

Patients with heart failure have comparable illness burden and palliative care needs to those with cancer. However, few of them are offered timely palliative care. One main barrier is the difficulty in identifying those who require palliative care. Several palliative care needs-assessment/measurement tools were used to help identify these patients and assess/measure their needs, but it is not known which one is the most appropriate for this population. This review aimed to identify the most appropriate palliative care needs-assessment/measurement tools for patients with heart failure. Cochrane Library, MEDLINE Complete, AMED, PsycINFO, CINAHL Complete, EMBASE, EThOS, websites of the identified tools, and references and citations of the included studies were searched from inception to 25 June 2020. Studies were included if they evaluated palliative care needs-assessment/measurement tools for heart failure populations in terms of development, psychometrics, or palliative care patient/needs identification. Twenty-seven papers were included regarding nineteen studies, most of which were quantitative and observational. Six tools were identified and compared according to their content and context of use, development, psychometrics, and clinical applications in identifying patients with palliative care needs. Despite limited evidence, the Needs Assessment Tool: Progressive Disease – Heart Failure (NAT:PD-HF) is the most appropriate palliative care needs-assessment tool for use in heart failure populations. It covers most of the patient needs and has the best psychometric properties and evidence of identification ability and appropriateness. Psychometric testing of the tools in patients with heart failure and evaluating the tools to identify those with palliative care needs require more investigation.

**Electronic supplementary material:**

The online version of this article (10.1007/s10741-020-10011-7) contains supplementary material, which is available to authorized users.

## Introduction

Palliative care is defined by the World Health Organization (WHO) as “an approach that improves the quality of life of patients and their families facing the problem associated with life-threatening illness…” [1]. It is a team-based, holistic approach that aims to address the multidimensional needs of patients and families: physical, psychological, social, and spiritual [1]. The basic palliative care needs of patients are managed by the patient’s usual care team (for example, primary care practitioner, cardiologist, heart failure nurse), while more complex needs are managed by a multidisciplinary specialist team with extensive training in palliative care [2, 3].

Patients with heart failure have a significant symptom burden and palliative care needs [4, 5], which are comparable with those with cancer [6, 7]. Several guidelines call for integrating palliative care into standard heart failure management [8–10]. Providing palliative care to these patients results in an improvement in their physical and psychological symptoms, quality of life, and satisfaction; increase in documentation of care preferences; and decrease in the use of medical service [11–13]. Despite this, patients with heart failure have less access to palliative care than those with cancer, and most of their palliative care consultations occur late in their life [14]. There are many barriers to providing palliative care to patients with heart failure [15, 16]. One major barrier is the difficulty in identifying those who need palliative care [17].

Using structured research tools can aid in identifying patients with heart failure who need palliative care [18]. Generally, these tools fall in one of two categories: those predicting end of life (prognostic tools) and those assessing/measuring patient needs (needs-assessment/measurement tools) [18]. Given the unpredictable trajectory of heart failure, prognostic tools are of limited value for identifying patients with a high risk of mortality who can benefit from palliative care [19]. The National Institute for Health and Care Excellence (NICE) guidelines do not recommend their use to determine if patients with heart failure need palliative care referral [20]. These tools do not correlate strongly with the palliative care needs of heart failure populations [19] nor do they account for the improvement in their quality of life [21]. On the other side, tools that focus on assessing/measuring patient needs, instead of predicting prognosis, are more appropriate for the timely initiation of palliative care for patients with heart failure [18, 22]. These tools can identify patient needs early before evidence of poor prognosis [23], provide a systematic assessment/measurement of patients’ needs which are often underreported by patients or assessed/measured differently by healthcare professionals [24, 25], facilitate discussion with the care team, and elicit patient preferences and goals of care [26].

Despite their advantages, some challenges exist for the use of palliative care needs-assessment/measurement tools in heart failure populations. These tools require further evaluation to determine their ability to enhance the timely introduction of palliative care in these patients [18]. Furthermore, most of these tools have not been widely implemented and few have been specifically developed and validated for non-cancer conditions [27, 28]. Several factors should be taken into consideration when selecting the most appropriate palliative care needs-assessment/measurement tool, including the aim of assessment, target patients, patient capabilities, clinical settings, administration mode, and its psychometric and practicality properties [27]; the latter defined as the burden of completing the tool on respondents (acceptability) and administrators (feasibility) [29, 30].

The intended use of the tools is another important factor to guide the selection of appropriate tools [31]. While some tools are mainly used as screening instruments to identify patients who require palliative care based on their deteriorating health and potential palliative care needs (patient identification tools), others are primarily used to provide a more holistic evaluation of those unmet needs (needs identification tools) [32]. Furthermore, while some tools are designed to measure patient needs (needs-measurement tools), others are designed to assess these needs as clinical decision aids (needs-assessment tools) [33]. Needs-measurement tools enable screening, monitoring, and scoring patient needs over time to track changes in health status and evaluate the effectiveness and quality of provided care [34]. When used alone, these tools may not trigger healthcare professionals to act on the identified needs as they may lack the skills and knowledge to interpret the scores [35, 36]. Therefore, they may have little contribution to clinical decision-making on their own [37]. On the other hand, needs-assessment tools, as clinical decision aids, facilitate the evaluation of patient needs, assignment of actions to address those needs, and understanding of care options and outcomes [33, 38]. These tools are ideally used as adjuncts to patient counseling to assist healthcare professionals in making the most appropriate decisions on patient care [33]. They are not intended to be prescriptive or used as an endpoint in themselves, but rather as a support and starting point for patient-centered care [33].

Comparisons between palliative care needs-assessment/measurement tools used in heart failure populations are lacking. It is not known which tools are better for palliative care patient/needs identification and which have the best psychometric and practicality evidence in these patients. There are no systematic reviews to critique these tools in identifying patients with heart failure who have palliative care needs. Three systematic reviews demonstrated tools that could be used to identify palliative care patients in primary care settings [28, 32, 39]. However, these were not specific to heart failure populations and limited to one setting. Another review of palliative care needs-assessment tools used in patients with chronic heart failure was not systematic, nor did it compare the psychometric properties in detail [18]. A comprehensive comparison between palliative care needs-assessment/measurement tools used in heart failure populations is needed to determine the most appropriate tools for identifying patients who require palliative care and assessing/measuring their needs. Subsequently, these needs can be acted upon to improve patients’ quality of life.

### Review question

What are the most appropriate palliative care needs-assessment/measurement tools for use in patients with heart failure?

### Review objectives

1. Identify palliative care needs-assessment/measurement tools used to identify patients with heart failure who have palliative care needs.
2. Compare these tools regarding their content (included items, length, addressed need domains) and context of use (clinical settings, completion method).
3. Compare the development and intended use of the tools.
4. Compare the psychometric and practicality properties of the tools in patients with heart failure.
5. Compare the clinical applications of the tools in identifying patients with heart failure who have palliative care needs.

## Methods

The review protocol was registered with the International Prospective Register of Systematic Reviews (PROSPERO) on December 2018 under registration number CRD42018118376. Quantitative, qualitative, and mixed-methods studies were included in the review to maximize the evidence on using the tools in patients with heart failure, where limited research is available [40]. The review was written following the guidance of the adapted Preferred Reporting Items for Systematic Reviews and Meta-Analyses (PRISMA) for reporting systematic reviews of qualitative and quantitative evidence [41]. Covidence online software program was used to facilitate systematic review management.

### Inclusion/exclusion criteria

Studies were included if they met all these criteria:

- Included adults 18 years of age or older with a primary diagnosis of heart failure.
- Evaluated palliative care needs-assessment/measurement tools, defined as structured multi-item research instruments developed for identifying palliative care patients/needs.
- Evaluated more commonly used tools, defined as those which were used for identifying heart failure populations with palliative care needs in more than one study retrieved through the review search.
- Aimed to evaluate the tools in terms of development, psychometrics or practicality, or palliative care patient/needs identification.
- Primary empirical quantitative, qualitative, or mixed-methods studies where quantitative and qualitative data were combined at the stage of data collection and/or analysis.
- Published in English or Arabic.

Studies that evaluated guidelines, pathways, and individual items were excluded. Case reports, opinion pieces, editorials, commentaries, letters, retrospective studies, reviews, and secondary research were also excluded.

### Search strategy

A sensitive search strategy was applied to retrieve relevant studies and tools after consulting experienced librarians. Cochrane Library, MEDLINE Complete (EBSCO), AMED (EBSCO), PsycINFO (EBSCO), CINAHL Complete (EBSCO), and EMBASE (Ovid) were searched from inception to 25 June 2020. The following secondary resources were also searched: websites of the retrieved tools where available, EThOS for United Kingdom’s (UK’s) doctoral research theses, and citing and cited articles of the included studies. Search terms for *palliative care*, *heart failure*, and *tool* were combined in each database using both free-text terms and Medical Subject Headings (MeSH) where available (Table 1). The search strategy for EMBASE (Ovid) is presented in Supplemental Table 1. Duplicates were removed from the retrieved records using EndNote X8 and Covidence.

**Table 1:** Key search terms used in the review

Key search terms*	
Concept 1	Palliative care OR Terminal care OR Long-term care OR End of life care OR Hospice OR Advance care planning
Concept 2	Heart failure OR Cardiac failure OR Ventricular dysfunction OR Low cardiac output OR Dilated cardiomyopathy OR Congestive cardiomyopathy OR Cardiogenic shock
Concept 3	Tool OR Survey OR Questionnaire OR Checklist OR Inventory OR Scale OR Instrument OR Indicator OR Measure OR Index OR Model OR Criteria OR Calculator OR Score
Filters/limits	
Population	Human
Language	English or Arabic
Study design	Empirical research
Date	No limits
Settings	No limits

*These terms are not exhaustive. An example of a comprehensive search strategy for EMBASE (Ovid) is shown in Supplemental Table 1

### Study/tool selection

Titles and abstracts of retrieved studies were screened by the main author (BR). A second reviewer (IM) screened 10% of them independently. The agreement rate for the studies screened was 97% which demonstrated a high level of agreement. Full texts of potentially relevant studies were screened by BR to determine their eligibility, while IM screened 25% of those independently as the agreement rate was 80%. Discrepancies were resolved through discussion which helped identify screening issues and discuss the inclusion criteria. A third reviewer (AG or NP) was consulted when necessary.

### Data collection

Data extraction tables were created for the included studies. They were piloted first on a sample of studies and continuously amended until the final versions were developed. Extracted data included study design, objectives, population, settings, and country; method of and reason for tools’ development; results of psychometric and practicality testing; method of identifying patients requiring palliative care and their needs; and results of tools’ applications in palliative patient/needs identification. Relevant data were extracted from the included papers by BR. IM extracted data from about half of the papers independently. All disagreements were resolved through discussion which helped identify extraction issues and refine the data extraction tables. There was no need to refer to the third reviewer. First authors of the included studies were contacted by email to clarify vague information if necessary, and all of them responded. Data were also extracted from the tools themselves and their associated guides if available. Extracted data included primary instruments from which the tools were adapted, settings of use, completion method and time, and involved items and need domains. The latest edition/version of each tool at the time of synthesizing the evidence was compared with the others.

### Criteria to assess tools’ psychometrics and practicality

The psychometric and practicality properties of the included tools were assessed by BR using the Oxford Patient-Reported Outcome Measures (PROMs) Group criteria for selecting PROMs in clinical trials [31]. Although the tools in this review were not all PROMs, this seemed the most appropriate tool to use as it provides detailed guidance on how to assess each of these criteria. Among the eight criteria suggested by the Oxford PROMs Group, the five which have been more often used and cited on standard checklists and discussions were compared: Acceptability, Feasibility, Reliability, Validity, and Responsiveness [31].

### Quality appraisal

To assess the quality of the heterogeneous studies (quantitative, qualitative, and mixed methods), Hawker et al.’s tool for appraising disparate data was used [42]. This instrument assesses the quality of studies based on nine criteria which can be scored from one (very poor) to four (good). The minimum and maximum possible scores per study are nine and 36, respectively. The methodological quality of the included studies was described and considered in the synthesis stage. Studies were not excluded based on their methodological rigor or assigned scores. Quality assessment of the included papers was performed by BR, while IM assessed the quality of about half of them independently. Disagreements were resolved through discussion which helped identify quality appraisal issues and critique the studies more thoroughly. There was no need to refer to the third reviewer.

### Synthesis method

Narrative synthesis, guided by Popay et al.’s framework, was used to synthesize the findings from the heterogeneous studies [43]. Tools were described narratively, and studies were tabulated and grouped according to the evaluated tool and their application to discover patterns within and across the groups. Subsequently, relationships were explored within and between the studies. The synthesis process was then critiqued where the limitations of the synthesis methodology, influence of low-quality studies on the synthesis results, made assumptions, and areas for future research were highlighted. Synthesizing the evidence from the included studies was carried out by BR.

## Results

### Study selection

**Fig. 1:**
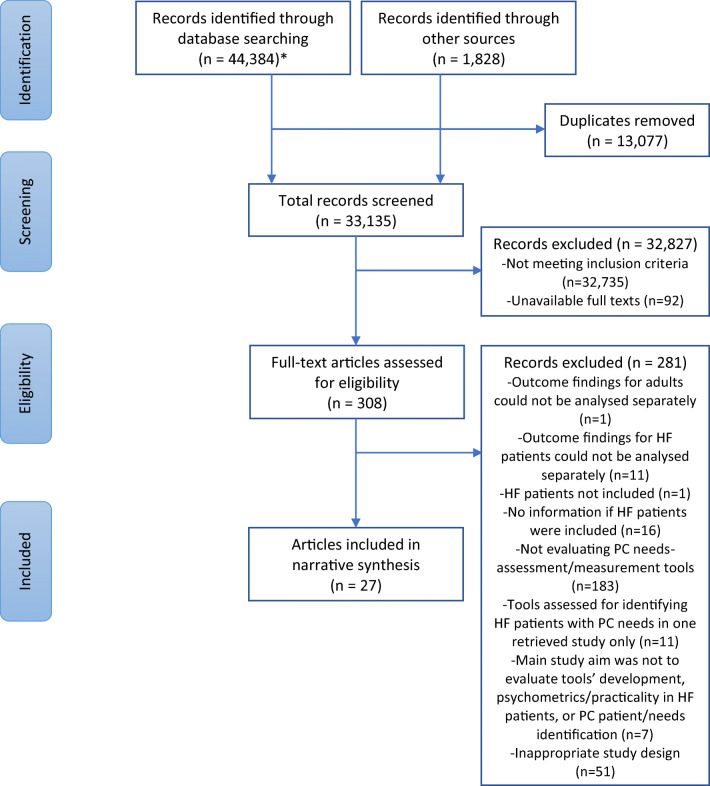
PRISMA flow diagram of study selection. HF heart failure, PC palliative care. *Cochrane Library, MEDLINE Complete, AMED, PsycINFO, CINAHL Complete, and EMBASE were originally searched from inception to 4 January 2019. The latest search update was run in these databases on 25 June 2020 except for CINAHL Complete because of end of subscription

The search strategy for the primary and secondary resources retrieved a total of 46,212 records, which were reduced to 33,135 after removing duplicates. The titles/abstracts of these papers were screened for relevance and meeting the inclusion criteria, resulting in 308 papers for full-text screening. Among these, 27 papers were included in the review about 19 studies. The included studies differ in their design: ten were quantitative [19, 44–54], one qualitative [55], and eight of mixed-methods design [26, 56–68]. All studies were observational except for one interventional study [61–64], one pilot study [59], and one feasibility study [26, 56]. The PRISMA flow diagram of study selection is presented in Fig. 1 [69].

The included papers were classified into three categories based on how the included tools were evaluated: development studies, psychometrics/practicality studies, and palliative care patient/needs identification studies (identification studies) (Table 2). Some studies fitted into more than one category as they were used for more than one purpose. There were five development studies, five psychometrics/practicality studies, and 17 identification studies. Quality scores of studies ranged from 22 to 35 with a median of 29, indicating moderate to good quality.

**Table 2:** List of the included tools and corresponding evaluation studies with their overall quality scores using Hawker et al.’s tool

Tool	Development study	Quality score*	Psychometrics/practicality study^#^	Quality score*	Identification study	Quality score*
IPOS	Schildmann et al. [55]	32	Kane et al. [56]	29	Kane et al. [56] Kane et al. [26] (follow-up paper)	29 30
			Roch et al. [57]	28	Roch et al. [57]	28
GSF-PIG	--	--	--	--	Milnes et al. [44]	27
					Haga et al. [19]	30
					Gardiner et al. [46] Ryan et al. [45] (follow-up paper)	28 30
					Pandini et al. [47]	24
RADPAC	Thoonsen et al. [60]	27	--	--	Thoonsen et al. [61] (protocol) Thoonsen et al. [62] Thoonsen et al. [63] (follow-up paper) Thoonsen et al. [64] (follow-up paper)	NA 32 32 29
SPICT	Highet et al. [65]	27	--	--	Highet et al. [65]	27
					Hamano et al. [48]	26
					Hamano et al. [49]	29
NAT:PD-HF	Waller et al. [58]	30	Waller et al. [58]	30	Waller et al. [58]	30
			Janssen et al. [59]	35	Janssen et al. [59]	35
			Campbell et al. [51]	28	Campbell et al. [50] (protocol) Campbell et al. [51]	NA 28
NECPAL	Gómez-Batiste et al. [66]	24	--	--	Gómez-Batiste et al. [66] Gómez-Batiste et al. [67] (follow-up paper) Amblàs-Novellas et al. [68] (follow-up paper)	24 30 29
					de-la-Rica-Escuín et al. [52]	30
					Orzechowski et al. [53]	23
					Gastelurrutia et al. [54]	22

*NA* not applicable. These papers are study protocols with no results to critique and therefore could not be assigned a total score in Hawker et al.’s tool

*Scores are out of 36

^#^Some studies in this column were not designed to test psychometrics/practicality but some data on these aspects were indirectly provided

### Identifying palliative care needs-assessment/measurement tools used to identify patients with heart failure who have palliative care needs

Several tools were found that had been or could be used for identifying patients with heart failure who require palliative care. Among these, six palliative care needs-assessment/measurement tools were identified as per the inclusion criteria and compared:

1. Integrated Palliative care Outcome Scale (IPOS) [55]
2. Gold Standards Framework - Proactive Identification Guidance (GSF-PIG) [70]
3. Radboud Indicators for Palliative Care Needs (RADPAC) [60]
4. Supportive and Palliative Care Indicators Tool (SPICT) [65]
5. Needs Assessment Tool: Progressive Disease - Heart Failure (NAT:PD-HF) [58]
6. Necesidades Paliativas - Palliative Needs (NECPAL) [66]

### Comparing the tools regarding their content and context of use

The main features and comparisons of the tools are displayed in Table 3. All tools were based on previous tools that informed their development except RADPAC, which was informed by extracting indicators used for identifying patients with palliative care needs from the literature [60]. Some tools were derived from each other which explains their similarities.

**Table 3:** Main features and comparisons of the tools

Tool	IPOS (version 1)	GSF-PIG (6th edition, 2016)	RADPAC (original)	SPICT (April 2019)	NAT:PD-HF (original)	NECPAL (version 3.1, 2017)
Main tools from which the tool was adapted	POS, POS-S, APCA African POS	NHPCO tool	--	NHPCO tool, GSF-PIG, PPS, PPI	PC-NAT	GSF-PIG, SPICT
Generic vs HF-Specific	Generic	Generic	Generic	Generic	HF-Specific	Generic
Clinical settings						
Diseases for which the tool can be used	Multiple (including heart failure)	Multiple (including heart disease)	Multiple (cancer, congestive heart failure, COPD)	Multiple (including heart/vascular disease)	Chronic heart failure	Multiple (including chronic heart disease)
Clinical settings for tool use	Multiple	Multiple	Primary care/general practice	Multiple	Multiple	Multiple
Completion method						
Completed by	Healthcare professionals (staff version), patients (patient version)	Healthcare professionals	Primary care practitioners	Healthcare professionals	Healthcare professionals	Healthcare professionals
Objective vs subjective*	Subjective	Objective, subjective	Objective, subjective	Objective, subjective	Subjective	Objective, subjective
Items						
Surprise Question	X	✓	X	X	X	✓
General indicators of health decline or PC need	X	✓	X	✓	X	✓
Disease-specific indicators of health decline or PC need	X	✓	✓	✓	X	✓
Open questions	✓	X	X	X	X	X
Length						
Number of items (for HF patients)	17 (+ 2 open questions)	17	7	9	20	18
Average time for completion	Staff version: 2–5 min Patient version: 8 min	--	--	-- (Older versions: 5–7.5 min)	5–10 min. (Dutch version: 26 min)	-- (Older version: 2–8 min)
Minimal criteria to identify HF patients who require PC	--	SQ+, or general indicators, or two HF-specific indicators	--	Any general indicator or the HF-specific indicator	--	SQ+ plus any other parameter
Need domains						
Physical	✓	✓	✓	✓	✓	✓
Psychological	✓	✓	X	X	✓	✓
Social	✓	X	X	X	✓	✓
Spiritual	✓	X	X	X	✓	X
Others	Informal carer, information, financial/personal	--	--	Informal carer	Informal carer, information, financial/legal, treatment regimens	--

*APCA*, African Palliative Care Association; *COPD*, Chronic Obstructive Pulmonary Disease; *HF*, heart failure; *NHPCO*, National Hospice and Palliative Care Organization; *PC*, palliative care; *PC-NAT*, Palliative Care-Needs Assessment Tool; *POS*, Palliative care Outcome Scale; *POS-S*, Palliative care Outcome Scale-Symptoms; *PPI*, Palliative Prognostic Index; *PPS*, Palliative Performance Scale. SQ+: a negative answer to the Surprise Question (healthcare professionals would not be surprised if the patient dies within the next year)

*Objective: medical records. Subjective: clinical judgement or patient/informal caregiver input

#### Included items

The tools include different items to identify patients with palliative care needs. GSF-PIG and NECPAL include the surprise question (*would you be surprised if the patient dies in next year?*) as the first step for identification [71], followed by general and disease-specific indicators of health decline. SPICT does not have the surprise question but includes general and disease-specific indicators, while RADPAC has only disease-specific indicators. In all these tools, a set of indicators specific to heart failure, or heart disease, exists. On the other hand, IPOS and NAT:PD-HF do not have indicators for patient identification. Instead, they include items that evaluate a variety of patient needs. IPOS consists of open-ended questions about patient main problems and unlisted symptoms alongside closed-ended questions on patient and caregiver needs which are answered using a Likert scale. It provides a total score whichprovides measurement of the overall patient needs. NAT:PD-HF consists of four sections that address patient and caregiver needs: *priority referral for further assessment*, *patient wellbeing*, *caregiver/family ability to care for patient*, and *caregiver wellbeing*. Needs identified in the last three sections can be rated according to their significance: *none*, *some/potential*, and *significant*. Moreover, actions are suggested for these needs: *direct management by the healthcare professional*, *management by another care team member*, and *referral to members outside the team*.

#### Clinical settings

Only NAT:PD-HF is specific for use in patients with heart failure [58]. All other tools can be used in multiple diseases. RADPAC was developed for use in primary care [60], while the other tools can be used in different healthcare settings.

#### Completion method

Other than IPOS which has a version for staff completion and another for patient completion, all tools were designed to be completed by healthcare professionals with interaction from patients or informal caregivers. All tools have a subjective element of completion where healthcare professionals use their clinical judgement (for example, *to assess symptoms severity or health decline*) or where patients/caregivers provide their input (for example, *to request for palliative care or rate their symptoms*). Furthermore, GSF-PIG, RADPAC, SPICT, and NECPAL require information from patients’ medical records such as the number of hospitalizations and weight.

#### Length

The length of tools varies with a range of seven items for completion (RADPAC) to 20 items (NAT:PD-HF). IPOS and NAT:PD-HF contain more items than other tools and although they may take longer to complete, they provide a more comprehensive evaluation of patient needs. SPICT and NECPAL need less than 8 min to fill [72–74]. IPOS patient version takes about 8 min for completion while the staff version takes about 2 to 5 min [75]. NAT:PD-HF needs about 5 to 10 min [27], although its Dutch translation needed an average of 26 min to be completed by heart failure nurses who were untrained in palliative care [59].

#### Addressed need domains

NAT:PD-HF covers more palliative care needs than any other tool, including the key need domains advocated by the WHO: physical, psychological, social, and spiritual [1]. It is the only tool that asks if patients have issues in managing their medication and treatment regimens. IPOS is also comprehensive and addresses most of the need domains contained in NAT:PD-HF. NECPAL misses the spiritual issues, while GSF-PIG, RADPAC, and SPICT address mainly the physical symptoms of patients.

In summary, NAT:PD-HF and IPOS outweigh other tools regarding the content and context of use. Both can be used in multiple clinical settings, completed in a reasonable time frame without reviewing patient medical records, provide a comprehensive assessment/measurement of patient and informal caregiver needs, and address more palliative care needs than other tools. Compared with NAT:PD-HF, IPOS has a patient version for completion which can decrease staff burden, includes open questions which enable patients to outline their main problems and unlisted symptoms, and requires less time for filling. However, unlike NAT:PD-HF, IPOS does not explicitly address treatment complexity among patient needs, neither does it have a correspondent action to be taken for the identified concerns.

### Comparing the development and intended use of the tools

None of the tools was originally developed for use in patients with heart failure. Only NAT:PD-HF was adapted specifically for use in these patients from a similar tool for patients with cancer [58]. All other tools are generic but have been used for patients with heart failure. A heart failure specific version of IPOS has not been formally tested yet [76]. All tools were developed in high-income countries, and half of them (IPOS, GSF-PIG, SPICT) were developed in the UK. The clinical expertise of healthcare professionals contributed to tools’ development. Similarly, literature reviews were conducted to aid in the development of all tools except GSF-PIG [70]. Interestingly, all tools have an original development paper except GSF-PIG. In conclusion, GSF-PIG underperforms compared with other tools in this comparison aspect.

GSF-PIG, RADPAC, SPICT, and NECPAL were developed to identify patients who require palliative care (*patient identification tools*) [60, 65, 66, 70], while IPOS and NAT:PD-HF were developed to provide a more comprehensive evaluation of the palliative care needs of patients (*needs identification tools*) [55, 58]. The patient identification tools were mainly developed as *clinical decision aids* which can be used during patient consultation to decide whether patients require palliative care and subsequently to prompt more holistic needs-assessment/measurement. SPICT, for example, is recommended to be used alongside IPOS to get a more complete picture on patient needs [77]. IPOS, on the other hand, was developed as an *outcome measure* to identify and score patient symptoms and concerns. It does not provide recommendations on how to address the identified needs and thus, clinical decision support tools are needed to interpret its scores [33]. NAT:PD-HF is not an outcome measure. It is mainly used as a clinical decision aid during patient consultation to classify the level of concern (none, some, significant) and triage actions for each identified need (managed directly, managed by other care team member, referral required). The main purpose and intended use of the tools are summarized in Table 4.

**Table 4:** Main purpose and intended use of the tools

Tool*	IPOS (version 1)	GSF-PIG (6th edition, 2016)	RADPAC (original)	SPICT (April 2019)	NAT:PD-HF (original)	NECPAL (version 3.1, 2017)
Patient identification		✓	✓	✓		✓
Needs identification	✓				✓	
Needs assessment/decision aids		✓	✓	✓	✓	✓
Needs measurement	✓					

*This classification should not be considered rigid as there can be some overlap in these applications

### Comparing the psychometric and practicality properties of the tools in patients with heart failure

In the general population, IPOS and SPICT have the best evidence of validity, reliability, and practicality [55, 65, 72, 73, 78–84], followed by NECPAL and RADPAC [60, 66], while no formal validation studies were found for GSF-PIG. Still, the psychometric and practicality properties of the tools were rarely assessed in heart failure populations (Table 5). Only NAT:PD-HF (Original NAT:PD-HF), its Dutch translation (Dutch NAT:PD-HF), IPOS (Original IPOS), and its German translation (German IPOS) had their practicality properties tested in these patients [56–59]. Besides, only Original NAT:PD-HF and Dutch NAT:PD-HF had some of their psychometric properties tested in this population [51, 58, 59].

**Table 5:** Psychometric and practicality properties of the tools in patients with heart failure, using the Oxford Patient-Reported Outcome Measures Group criteria

Tool	Acceptability	Feasibility	Reliability	Validity	Responsiveness
IPOS (original, patient version 1, 7-day recall)	+++	+++	0	0	0
IPOS (Gemran, patient version, 3-day recall)	++	0	0	0	0
GSF-PIG	0	0	0	0	0
RADPAC	0	0	0	0	0
SPICT	0	0	0	0	0
SPICT (Japanese, SPICT-J)	0	0	0	0	0
NAT:PD-HF (original)	+	+++	+	++	0
NAT:PD-HF (Dutch)	–	–	0	–	0
NECPAL	0	0	0	0	0

– = evidence does not support criteria

0 = not reported or no evidence in favor

+ = some limited evidence in favor

++ = some good evidence in favor, but some aspects do not meet criteria or some aspects not reported

+++ = good evidence in favor

#### Acceptability

Acceptability of the tools to patients was only tested for Original NAT:PD-HF, Dutch NAT:PD-HF, Original IPOS, and German IPOS. Although acceptability of NAT:PD-HF versions was not directly assessed from the perspective of patients, it was assessed using other parameters such as *time to complete* and *translation and cultural applicability* [31]. Overall, both IPOS versions and Original NAT:PD-HF were acceptable, with more evidence in favor of IPOS [56–58]. On the contrary, Dutch NAT:PD-HF had negative evidence of acceptability [59].

#### Feasibility

Feasibility of the tools for healthcare professionals was only tested for Original NAT:PD-HF, Dutch NAT:PD-HF, and Original IPOS. While Original IPOS and Original NAT:PD-HF were feasible (easy to complete in a short time) [56, 58], Dutch NAT:PD-HF had negative evidence of feasibility [59].

#### Reliability

Reliability was only assessed for Original NAT:PD-HF [58]. Results of testing inter-rater reliability showed good agreement between the raters for each tool item. Internal consistency and test-retest reliability were not tested.

#### Validity

Validity was only assessed for Original NAT:PD-HF and Dutch NAT:PD-HF. Original NAT:PD-HF showed good face, content, and concurrent (construct) validity [51, 58]. Construct validity was tested in one study by identifying the correlation between the items in the NAT:PD-HF *patient wellbeing* section and corresponding items from the Heart Failure Needs Assessment Questionnaire (HFNAQ) [58]. In another study which was not designed to test the tool psychometrics, a statistically significant relationship was found between having a *significant* concern on any item in the NAT:PD-HF *patient wellbeing* section and the construct of specialist palliative care needs as defined by the authors (persistently severe impairment of any of four PROMs without improvement, or severe impairment immediately preceding death) (*p* = 0.008) [51]. The other tool sections were not tested for construct validity in both studies. In contrast to Original NAT:PD-HF, Dutch NAT:PD-HF showed poor construct and criterion validity [59]. These were tested by identifying the correlation between some items of Dutch NAT:PD-HF and three outcome measures: Dutch Edmonton Symptom Assessment System (ESAS), Australia-modified Karnofsky Performance Scale (AKPS), and Family Appraisal of Caregiving Questionnaire for Palliative Care (FACQ-PC). Of note, the evaluating study was a pilot study and not designed to test the tool’s validity.

#### Responsiveness

Responsiveness was not evaluated for any tool.

In conclusion, Original NAT:PD-HF is the most extensively tested and psychometrically robust tool in heart failure populations. It is the only tool validated in this population and has some evidence of reliability. Also, it is feasible for healthcare professionals and has some evidence of acceptability to patients. Although IPOS has more acceptability evidence than NAT:PD-HF, its psychometrics has not been tested in heart failure populations. Psychometrics and practicality of the other tools were not tested at all in this population.

### Comparing the clinical applications of the tools in identifying patients with heart failure who have palliative care needs

The characteristics of the identification studies are shown in Supplemental Table 2. Detailed results of the tools’ applications in identifying heart failure populations with palliative care needs are presented in Supplemental Table 3.

#### Breadth of tools’ application in heart failure populations

Few identification studies were found for each tool. GSF-PIG and NECPAL were the most commonly evaluated (four studies each) [19, 44–47, 52–54, 66–68], followed by SPICT and NAT:PD-HF [48–51, 58, 59, 65] (three studies each), IPOS (two studies) [26, 56, 57], and lastly RADPAC (one study) [61–64]. GSF-PIG was evaluated in more countries than other tools (four countries), followed by NAT:PD-HF (three countries). NECPAL was evaluated in diverse healthcare settings, while IPOS, GSF-PIG, SPICT, and NAT:PD-HF were evaluated for inpatients and outpatients. More patients with heart failure were screened by NAT:PD-HF and NECPAL compared with other tools. Baseline data for the tools-screened patients were described in more detail in NAT:PD-HF and IPOS studies. While NAT:PD-HF was evaluated for several types and classes of heart failure and was the only tool evaluated for those with acute on chronic heart failure, patients who lacked the cognitive capacity to participate or consent were excluded from its studies.

#### Use for palliative care patient/needs identification

All tools were used to identify palliative patients (patient identification) and evaluate their needs (needs identification) except RADPAC which was mainly applied by the authors to identify palliative patients [61–63]. When used for patient identification, GSF-PIG (in one study) and RADPAC were combined with a more comprehensive needs-assessment/measurement tool [45, 46, 61, 62].

#### Ability and appropriateness of the tools for palliative care patient/needs identification

The proportion of patients with heart failure identified by the tools for palliative care among those screened was considered an indicator of their *identification ability*. This could not be calculated in many studies because of missing or vague data and the lack of a clear gold standard of what a palliative care patient is. RADPAC-trained primary practitioners identified only 6% of patients with heart failure in a randomized controlled trial [62]. One year after training, these trained practitioners did not identify any patient, while those untrained identified more patients shortly after RADPAC administration [63]. SPICT identified only a few patients with heart failure although the proportion in one study was misleadingly high because of the small sample size [48]. GSF-PIG identified 86% of patients with heart failure in one study [19], while NECPAL identified 32%, 55%, and 91% in three studies [53, 54, 67]. IPOS and NAT:PD-HF identified 56% and 26% of patients with heart failure for specialist palliative care, respectively [51, 57]. NAT:PD-HF identified 100% of patients for palliative care in another study [59].

The baseline health characteristics and morbidity outcomes of idenitified patients were considered an indicator of the *appropriateness of identification* by the tools. However, this was not reported in most studies. The tool is robust if the patients it identified for palliative care had evidence of poor health. Poor health at baseline, evidenced by poor scoring in patient outcome measures, long or frequent hospitalizations, old age, and/or New York Heart Association (NYHA) class III-IV, was shown for many patients identified by IPOS [57], GSF-PIG [19, 45, 46], NAT:PD-HF [51, 59], and NECPAL [53, 54]. Likewise, better health at baseline, evidenced by NYHA class I–II, was observed in many patients who reported few significant psychological, social, and spiritual concerns in NAT:PD-HF [58]. Morbidity outcomes at follow-up periods of identified patients were only presented briefly in one GSF-PIG study, where identified patients did not have significantly more hospitalizations within a 1-year follow-up period as would have been expected [19].

#### Impact of the tools

Three tools were incorporated into palliative care interventions where healthcare professionals were trained on using the tools to identify, and subsequently act on, the palliative care needs of patients [56, 59, 61, 62]. IPOS, RADPAC, and Dutch NAT:PD-HF had no significant positive impact on patients with heart failure or their informal caregivers. The IPOS-based intervention resulted in mild improvement in the quality of life, symptom burden, and depression, though this was often transient and got worse at further follow-up periods [56]. Similarly, symptom burden, physical functioning, care dependency, and caregiver burden were not significantly improved after the Dutch NAT:PD-HF intervention and health status got significantly worse [59]. Additionally, it did not influence the recording of advance directives or hospital and emergency room visits. Of note, the studies that evaluated the intervention effect of IPOS and Dutch NAT:PD-HF were pilot/feasibility studies and not designed to test their effectiveness [56, 59]. In contrast, the RADPAC intervention effect was evaluated in a cluster randomized controlled trial where primary care practitioners used the tool to identify patients with palliative care needs [61, 62]. RADPAC intervention did not result in a significant difference between deceased patients of RADPAC-trained practitioners and those of untrained practitioners in the number of contacts with out of hours primary care service (primary outcome measure), contacts with own primary care practitioner, hospitalizations, and place of death (secondary outcome measures). In a post hoc analysis, identified patients from the trained group (only two with heart failure) had significantly better secondary outcome measures compared with all other patients, but the primary measure was not different.

#### Perspectives of healthcare professionals and patients on using the tools for identification

The three interventions described above were followed by interviews with healthcare professionals and/or patients to evaluate their perspectives on using the tools for identification [26, 59, 64]. The emerged themes were mainly positive for IPOS and RADPAC and negative for Dutch NAT:PD-HF. A common positive theme on IPOS and RADPAC was the identification of palliative needs (IPOS) and patients (RADPAC), though identifying those with heart failure was considered difficult by RADPAC. Dutch NAT:PD-HF was not found helpful to communicate about palliative care, while IPOS was found to facilitate patient-nurse communication although many patients did not consider it to have any clinical effect. Patient perspectives were only evaluated for IPOS while healthcare professionals were interviewed in all studies.

In summary, NAT:PD-HF outperformed other tools in the clinical applications in palliative patient/needs identification though this needs further testing. NAT:PD-HF has relatively wide application in heart failure populations and it was used for both patient identification and needs identification. NAT:PD-HF was able to identify high proportions of patients with heart failure who have palliative care needs and most importantly those identified had poor health at baseline, indicating a proper identification. The original NAT:PD-HF was not incorporated into an intervention in contrary to its Dutch translation. Like IPOS and RADPAC, Dutch NAT:PD-HF lacked a significant positive impact on patients/informal caregivers. Unlike these two tools, healthcare professionals were not positive in their comments on Dutch NAT:PD-HF and they listed many barriers for its use.

## Discussion

This is the first systematic review that comprehensively compares palliative care needs-assessment/measurement tools used in patients with heart failure. The main review question was to determine the most appropriate palliative care needs-assessment/measurement tools for use in heart failure populations to inform clinical practice. Six tools were identified and compared according to their content and context of use, development, psychometrics and practicality, and applications in identifying patients with palliative care needs. Based on the limited available evidence, NAT:PD-HF is the most appropriate palliative care needs-assessment tool for heart failure populations, though more studies are needed to confirm this. IPOS is promising and shares many advantages of NAT:PD-HF but it is less commonly studied in this population. Generalizability of the review results is limited by the small number of tool-evaluating studies and the heterogeneity of populations, interventions, outcomes, and health settings.

The results of this review are concordant with the recent European Association for Palliative Care (EAPC) position statement where a comprehensive palliative care needs-assessment tool was suggested to identify patients with unmet needs [9]. NAT:PD-HF, being validated for patients with heart failure, was suggested as an example of such a tool but this was not based on detailed comparisons with other tools. IPOS was also suggested as a trigger to initiate palliative care but categorized separately as a symptoms-assessment tool. SPICT was considered a patient identification tool that does not detail individual needs. Although SPICT was recommended over other tools in one review to identify palliative patients, this was concluded for the general population in primary care, and neither NAT:PD-HF nor IPOS was included in that review [39].

NAT:PD-HF was not identified in three previous systematic reviews that looked for tools used to identify general populations with palliative care needs in primary care [28, 32, 39]. It was probably seen as a needs identification rather than a patient identification tool. Indeed, NAT:PD-HF was developed for identifying patient needs rather than screening patients who require palliative care, although it has been used for both purposes [51, 58, 59]. Another non-systematic review of palliative care needs-assessment in patients with chronic heart failure included NAT:PD-HF but it did not seek which tool is the most appropriate for this population [18].

The tools have different items to identify patients with palliative care needs, including the surprise question, indicators of deterioration, and reported symptoms and concerns. The potential use of the surprise question as a simple method for identifying patients with palliative care needs had been acknowledged [85, 86]. However, RADPAC developers did not recommend it to trigger end of life discussions [60], and although it was included in SPICT original versions, it was removed later. Apart from this question, the items of some tools (GSF-PIG, RADPAC, and SPICT) address mainly patient physical symptoms. These tools may not be able to identify relatively asymptomatic patients with a high risk of dying [87, 88]. Therefore, a more comprehensive needs-assessment/measurement tool like NAT:PD-HF or IPOS would be more appropriate to use in this population.

The length of time to complete the tools should be accounted for to prevent staff/patient burden [27]. Reasons for the differences between the tools in time for completion include the tool purpose, number of items, and completion method [27, 39]. NAT:PD-HF and IPOS aim to identify the multidimensional palliative care needs of patients and hence, they have the largest number of items to complete. All items require clinical judgement or patient/informal caregiver input which may increase completion time [39]. The *action taken* section of NAT:PD-HF may contribute to the longer time needed to fill the tool compared with IPOS, but it may also prompt staff to think about how to act on the identified needs. IPOS does not have such section and it may just be filled and filed without having a clinical effect [26]. IPOS patient version (PROM) can be used outside the consultation time where each question is answered to provide a score measure for each concern and symptom. Conversely, although NAT:PD-HF takes a relatively few minutes to complete the form itself, it represents information obtained throughout a longer clinical assessment. This may explain the long time needed to complete its Dutch translation (26 min) [59]. Interestingly, the original cancer version of NAT:PD-HF (NAT:PD-C) did not prolong the average consultation time (18 min) indicating that the tool items are normally evaluated during consultations [89]. The other tools (GSF-PIG, RADPAC, SPICT, and NECPAL) are clinical consultation aids, like NAT:PD-HF, but they require screening medical records in addition to subjective judgements. No data about time for completion were available on the latest version of these tools at the time of synthesizing the evidence.

Regarding tools’ development, GSF-PIG, SPICT, and NECPAL were derived from prognostic tools but the focus has been shifted from determining prognosis to assessing needs for recognizing eligible patients for palliative care. This is supported by the results of a study where a high level of need was observed among patients identified by GSF-PIG although few of them died within a 12-month follow-up period [19]. Indeed, GSF-PIG was renamed from *Prognostic Indicator Guidance* to *Proactive Identification Guidance* although the tool content only showed minimal changes [70]. Likewise, the aim of SPICT was changed from “identifying people at risk of deteriorating and dying” to “identify people whose health is deteriorating [and] assess them for unmet supportive and palliative care needs…” [77]. Despite these endeavors, these tools are still used to determine prognosis which informs patient eligibility for palliative care [19, 78, 90].

The tools are not necessarily mutually exclusive; indeed, they can be used for different, and possibly complementary, purposes. One scenario is the use of one tool to screen for patients who require palliative care (patient identification), followed by another tool to evaluate their needs more comprehensively (needs identification) [32]. In this case, the patient identification tool provides a quick snapshot of patient needs, while the needs identification tool provides a more complete picture and holistic evaluation of these needs [27]. Another scenario is the use of one tool to measure general patient needs over time and another tool to identify specific needs and triage action to meet those needs [91]. IPOS, as a generic outcome measure which provides a total score and individual scores of patient needs, could provide a general summary of patient needs which could be then assessed in more detail using the heart failure specific tool NAT:PD-HF by determining the level of concern for each need and assigning actions to address those needs. Another possible use of the tools is to identify patients with specialist palliative care needs to be included in a randomized controlled trial of specialist palliative care versus standard care [92]. Providing a specialist palliative care intervention to those identified to have specialist palliative care needs is necessary to avoid diluting the effect size. This issue is common in heart failure research where patients with specialist palliative care needs are not differentiated from patients without these needs.

Given that the tools serve different purposes, their psychometric properties are not directly comparable. Nonetheless, no tool had been tested as widely as NAT:PD-HF. Original NAT:PD-HF has good validity and inter-rater reliability and was acceptable to staff and patients [51, 58]. The poor psychometric and practicality properties of Dutch NAT:PD-HF have several possible reasons [59]. Firstly, although the tool was translated using a forward-backward procedure, cultural adaptation was not adopted upon translation. Cultural adaptation is needed when a tool is used in another country and language to maintain its content validity [93], and poor translation may create an inequivalent tool to the original one [93, 94]. Secondly, the evaluating study was not designed as a primary psychometric study and its focus was not to test construct and criterion validity. Nonetheless, the correlation between some Dutch NAT:PD-HF items and three outcome measures was examined in an exploratory secondary analysis, and the results provided information on both validity types. Thirdly, the small sample size was a contributor to the lack of relationship between the constructs. Lastly, the heart failure nurses who administered the tool to patients lacked skills, knowledge, training, and experience in palliative care which led to difficulties in understanding the tool questions. This suggests that implementation issues may affect the tools’ ability to identify patient needs.

Two approaches were suggested in this review to evaluate the tools in identifying patients with palliative care needs. The first approach is to assess their identification ability by calculating the proportion of identified palliative patients (the more patients identified, the better is the tool). It was noted that a high proportion of identified patients may not always reflect a good tool’s identification ability. Proportions may be misleadingly high or low if the tool is used by untrained or unskilled staff or if few patients are screened [48, 49]. Also, a low proportion may reflect less severe disease rather than weak identification ability. Therefore, a better approach to evaluate the tools is to assess the appropriateness of identification by evaluating the health status of identified patients. Issues with identification were suggested for RACPAC and NECPAL. RADPAC-trained primary care practitioners identified a few patients for palliative care [62], most likely because the tool covers only physical patient needs so it could not identify those with psychosocial and spiritual needs. For NECPAL, more than 90% of patients with a negative answer to the surprise question were identified by the tool across all the evaluating studies [52–54, 67], which may suggest a little added value of the detailed NECPAL compared with the surprise question alone.

The lack of intervention effect of IPOS and Dutch NAT:PD-HF on health outcomes has many possible reasons [56, 59]. Firstly, the evaluating studies were not designed to test effectiveness. Secondly, worsening of health status over time is expected in patients with heart failure [8]. Without a control group, it is not possible to see a signal of benefit over time; deterioration may have happened faster without the intervention. Lastly, the actions taken by the nurses to address the identified patient needs might be inappropriate as they were not offered clinical guidelines on how to act upon the results of the tools. The interviews with heart failure nurses and patients after the IPOS intervention revealed that it could not trigger nurses to act on the identified needs [26]. The several barriers listed for Dutch NAT:PD-HF by interviewed heart failure nurses indicate the improper translation of the tool and lack of palliative care knowledge among nurses [59]. For RADPAC intervention, the lack of significant effect was justified by the small proportion of identified patients and identifying practitioners [61, 62]. The difficulty in identifying palliative patients with heart failure as reported by the interviewed primary care practitioners after the intervention revealed a tool identification problem [64].

To be clinically relevant, palliative care needs-assessment/measurement tools should be successfully implemented in practice by healthcare professionals. Barriers to implementation include high workload of healthcare professionals and limited resources and capacities; lack of expertise, knowledge, education, and training about palliative care in heart failure; and lack of communication skills with patients and informal caregivers [32, 59]. Additional barriers adopted from similar discussions on implementing advance care planning in heart failure care, where needs-assessment is a key element [95], exist on different levels. These include lack of support at the health system and institutional level; lack of an electronic information-recording and exchange system; lack of public education about palliative care; fear of losing hope and causing concern if palliative care is discussed with patients and informal caregivers; lack of trust and a long relationship with patients and informal caregivers to enable palliative care discussions; unstable physical, cognitive, and emotional conditions of patients; emotional impact on healthcare professionals when discussing palliative care; misconception that palliative care discussions reflect treatment failure; and lack of collaboration between healthcare professionals and consensus on who should fill the tool and assess the needs [96–98]. It is essential to overcome these barriers because no matter how well-developed, valid, acceptable, and feasible the tools are, they would be ineffective in clinical practice if no attention is paid to implementation issues. Successful implementation of the tools would facilitate the timely identification of patients with palliative care needs and subsequent access to palliative care services [32].

### Strengths and limitations

This review adopted a systematic method to search for relevant evidence, screen retrieved studies and tools, extract data from included ones, assess their quality, and synthesize their findings. A broad search strategy was used to retrieve most of the relevant studies. The review was not restricted to quantitative or qualitative studies as both were sought. It was written following the adapted PRISMA reporting guideline to enhance transparency [41]. The choice of the most appropriate tools was based on comprehensive comparisons according to predetermined criteria. Although NAT:PD-HF was suggested as an example of a good needs-assessment tool in the EAPC statement and another review, this was not based on such comparisons [9, 18].

The review has some limitations. Firstly, tools were excluded if they were not developed for palliative care patient/needs identification or used for identifying heart failure populations with palliative care needs in a single study retrieved through the review search. Including these tools in the review could have altered its findings. Secondly, the second reviewer was only partly involved in study screening, data extraction, and quality appraisal. He was not involved in assessing the tools’ psychometric and practicality properties and synthesizing the evidence. Thirdly, the psychometric and practicality properties of the tools were assessed using the Oxford PROMs Group criteria although all tools, except IPOS patient version, were clinical decision aids rather than PROMs. Needs-assessment tools are distinct from needs-measurement tools and they have different, though overlapping, purposes; therefore, the psychometric approaches for each are not directly comparable. The purpose and method of validation differ between these tool types and the psychometric items of responsiveness, although not assessed, may not apply to clinical decision aids. Fourthly, despite adopting a sensitive search strategy, some studies and tools might be missed as with any systematic review. Studies published in non-English or non-Arabic languages were not searched, and few gray literature sources were sought. Indeed, palliative care and heart failure studies are difficult to retrieve because of their inconsistent terminology [99, 100]. The term *heart disease* was used in some included studies and this was assumed to be equivalent to *heart failure* unless indicated otherwise.

Methodological limitations include the subjective nature of narrative synthesis which may affect transparency and reproducibility [101], though this was mitigated by adapting Popay et al.’s framework [43]; lack of consensus on the best tool for concomitantly appraising quantitative, qualitative, and mixed-methods studies [102], though the commonly cited Hawker et al.’s tool was used; and assignment of a total quality score for each study which is not agreed by some researchers [42]. Studies were not excluded based on their quality score. However, excluding lower quality studies would not have changed the answer to the review question, especially that NAT:PD-HF and IPOS studies scored in the upper range of the scale and would not have been excluded.

### Implications for research, practice, and policy

The tools need further assessment of their psychometric and practicality properties in patients with heart failure. Further evaluation of the tools for identifying heart failure populations with palliative care needs is also needed. Future studies should include a larger number of patients, evaluate patients with different types of heart failure and in multiple health settings, and adequately report the baseline data and health outcomes for identified patients. Cultural adaptation should be included in the tools’ translation to create tools equivalent to the original ones. Healthcare professionals should be aware of the different roles that needs-assessment/measurement tools can play and consider combining them where appropriate. Until more data become available, they are advised to use NAT:PD-HF to identify heart failure populations with palliative care needs. This should be followed by acting to address these needs and consequently improve health outcomes. Policymakers should adopt a needs-based approach for identifying patients requiring palliative care and integrate needs-assessment/measurement tools into the practice of healthcare professionals. Particular attention should be paid to implementation issues to enhance the clinical effectiveness of the tools in practice.

## Conclusion

Six palliative care needs-assessment/measurement tools used in patients with heart failure were identified and compared according to their content and context of use, development, psychometrics and practicality, and applications in identifying palliative care patients and needs. The tools are not necessarily mutually exclusive as they may serve different purposes including patient identification, needs identification, needs-measurement, and needs-assessment (decision aids). Comparison results suggested that NAT:PD-HF is the most appropriate palliative care needs-assessment tool for use in heart failure populations. It covers most of the patient needs and has the best psychometric properties and evidence of identification ability and appropriateness. However, this conclusion is based on limited evidence. Four retrieved tools lack studies on their psychometric and practicality properties in heart failure populations, and one of these (GSF-PIG) even lacks a research development paper. Nevertheless, NAT:PD-HF is preliminarily recommended for use in patients with heart failure, but it requires further testing and validation. IPOS has some similar advantages to NAT:PD-HF but less evidence is available on its use in heart failure populations.

## Electronic supplementary material

ESM 1 (DOCX 43.9 kb)

## Data Availability

Not applicable.
