# Supplementary material for: Palliative care needs-assessment and measurement tools used in patients with heart failure: a systematic mixed-studies review with narrative synthesis
Source: Heart Fail Rev. 2020 Aug 3;26(1):137–55. doi: 10.1007/s10741-020-10011-7 (PMC7769784; doi:10.1007/s10741-020-10011-7)
Supplement: Supplementary file 1 — (DOCX 43.9 kb) [file 10741_2020_10011_MOESM1_ESM.docx]

Electronic Supplementary Material

# Article title

Palliative care needs-assessment tools used in patients with heart failure: a systematic mixed-studies review with narrative synthesis

# Journal

Heart Failure Reviews

# Authors

Bader Nael Remawi, Amy Gadoud, Iain Malcolm James Murphy, Nancy Preston

# Corresponding author

Bader Nael Remawi

Lancaster Medical School, Faculty of Health and Medicine, Lancaster University, Lancaster, LA1 4YG, UK

Email: b.remawi@lancaster.ac.uk

## **Supplemental Table 1** Search strategy for EMBASE (Ovid)

| Keywords | Search terms | Search number |
| --- | --- | --- |
| Palliative care | exp palliative therapy/ **OR** palliative nursing/ **OR** hospice/ **OR** hospice nursing/ **OR** exp terminal care/ **OR** terminal disease/ **OR** exp terminally ill patient/ **OR** "palliat*".ab,kw,ti. **OR** "hospice*".ab,kw,ti. **OR** dying.ab,kw,ti. **OR** (close adj6 (death or die* or dead)).ab,kw,ti. **OR** long term care/ **OR** ((long term or longterm) adj6 (care* or caring or ill* or disease*)).ab,kw,ti. **OR** ((end or final or late or last) adj6 (stage* or phase*)).ab,kw,ti. **OR** endstage.ab,kw,ti. **OR** (end adj6 life).ab,kw,ti. **OR** EOL.ab,kw,ti. **OR** EOLC.ab,kw,ti. **OR** (last adj6 year adj6 life).ab,kw,ti. **OR** LYOL.ab,kw,ti. **OR** living will/ **OR** (advance* adj6 (care* or caring or plan* or directive*)).ab,kw,ti. **OR** "living will*".ab,kw,ti. **OR** (terminal* adj6 (care* or caring or ill* or disease* or stage* or phase*)).ab,kw,ti. **OR** ((incurable or serious or severe or critical or advanced or progressive) adj6 (ill* or disease* or stage* or phase*)).ab,kw,ti. **OR** bereavement/ **OR** exp bereavement support/ **OR** exp grief/ **OR** treatment withdrawal/ **OR** "bereav*".ab,kw,ti. **OR** (grief or griev*).ab,kw,ti. **OR** (treat* adj6 (withhold* or withdraw*)).ab,kw,ti. | 33. 1 **OR** 2 **OR** 3 **OR** 4 **OR** 5 **OR** 6 **OR** 7 **OR** 8 **OR** 9 **OR** 10 **OR** 11 **OR** 12 **OR** 13 **OR** 14 **OR** 15 **OR** 16 **OR** 17 **OR** 18 **OR** 19 **OR** 20 **OR** 21 **OR** 22 **OR** 23 **OR** 24 **OR** 25 **OR** 26 **OR** 27 **OR** 28 **OR** 29 **OR** 30 **OR** 31 **OR** 32 |
| Heart failure | heart failure/ or cardiogenic shock/ or cardiopulmonary insufficiency/ or cardiorenal syndrome/ or exp congestive heart failure/ or exp diastolic dysfunction/ or forward heart failure/ or exp heart ventricle failure/ or exp heart ventricle overload/ or high output heart failure/ or exp systolic dysfunction/ **OR** exp congestive cardiomyopathy/ **OR** HF.ab,kw,ti. **OR** CHF.ab,kw,ti. **OR** ((heart or cardi* or myocard* or ventricular or ventricle*) adj6 (failure* or decompensat* or dysfunction* or insufficienc* or incompet* or shock*)).ab,kw,ti. **OR** paroxysmal dyspnea/ **OR** LVSD.ab,kw,ti. **OR** ((dilated or congestive) adj6 cardiomyopath*).ab,kw,ti. **OR** (cardiac output adj6 low).ab,kw,ti. **OR** heart failure.af. | 44. 34 **OR** 35 **OR** 36 **OR** 37 **OR** 38 **OR** 39 **OR** 40 **OR** 41 **OR** 42 **OR** 43 |
| Tools | clinical assessment tool/ **OR** questionnaire/ or open ended questionnaire/ or structured questionnaire/ **OR** health care survey/ **OR** exp health survey/ **OR** checklist/ **OR** exp attitude scale/ **OR** rating scale/ **OR** visual analog scale/ **OR** clinical indicator/ **OR** self report/ **OR** "tool*".ab,kw,ti. **OR** "questionnaire*".ab,kw,ti. **OR** "scor*".ab,kw,ti. **OR** "scale*".ab,kw,ti. **OR** "checklist*".ab,kw,ti. **OR** "indicator*".ab,kw,ti. **OR** "instrument*".ab,kw,ti. **OR** "measure*".ab,kw,ti. **OR** "survey*".ab,kw,ti. **OR** "profile*".ab,kw,ti. **OR** "status*".ab,kw,ti. **OR** "calculator*".ab,kw,ti. **OR** "criteria*".ab,kw,ti. **OR** "model*".ab,kw,ti. **OR** "inventor*".ab,kw,ti. **OR** (index* or indices*).ab,kw,ti. **OR** numeric rating scale/ **OR** psychological rating scale/ | 73. 45 **OR** 46 **OR** 47 **OR** 48 **OR** 49 **OR** 50 **OR** 51 **OR** 52 **OR** 53 **OR** 54 **OR** 55 **OR** 56 **OR** 57 **OR** 58 **OR** 59 **OR** 60 **OR** 61 **OR** 62 **OR** 63 **OR** 64 **OR** 65 **OR** 66 **OR** 67 **OR** 68 **OR** 69 **OR** 70 **OR** 71 **OR** 72 |
| *Combination* | -- | 74. 33 **AND** 44 **AND** 73 |
| *Human filter* | (exp animal/ or exp invertebrate/ or nonhuman/ or animal experiment/ or animal tissue/ or animal model/ or exp plant/ or exp fungus/) not (exp human/ or human tissue/) | 75.  76. 74 **NOT** 75 |
| *Language limit* | limit 76 to (arabic or english) | 77. |
| *Design limit* | limit 77 to (editorial or letter) | 78.  79. 77 **NOT** 78 |

## **Supplemental Table 2** Characteristics of the identification studies

| Author, year, setting | Study design | Patient inclusion/exclusion criteria | Participants | Screened HF patients (%) |
| --- | --- | --- | --- | --- |
| IPOS | | | | |
| Kane et al. 2017,  Kane et al. 2018  Multi-center;  Hospital clinics; Ireland | Parallel mixed-methods intervention feasibility study, including semi-structured interviews  **Intervention:**  Training HFNSs on patient-centered care and IPOS | **Inclusion:**  Patients ≥ 18 years; Fluency and literacy in English; Good cognitive function; Advanced disease (NYHA class II-IV with either systolic dysfunction/HFrEF, symptoms with HFpEF, or symptoms with HFmrEF) | Patients with CHF: HFrEF, HFpEF, HFmrEF; and NYHA II-IV  HF patients screened by the tool (n=25); completed the follow-up outcome measures (n=23); included in the interviews (n=18)  HFNSs included in the intervention and interviews (n=4) | **Mean age:**  76 years  **NYHA class:**  II (32%)  III (64%)  IV (4%)  **LVEF:**  HFrEF (40%)  HFmrEF/HFpEF (60%) |
| Roch et al. 2020  Single center; Hospital inpatient; Germany | Cross-sectional | **Inclusion:**  Adults; Heart failure diagnosis; Inpatients in the hospital cardiology department; Understanding German language  **Exclusion:**  Cognitive impairment; Substantial comprehension problems; Poor general condition | Patients with HF: NYHA II-IV  HF patients screened by the tool (n=100) | **Mean age:**  78.5 years  **NYHA class:**  II (4%)  III (65%)  IV (31%)  **LVEF:**  ≤ 40% (40%)  > 40% (58%) |
| GSF-PIG | | | | |
| Milnes et al. 2019  Single center;  Hospital inpatient;  Australia | Observational cohort | **Inclusion:**  Patients > 18 years; Admitted to acute hospital  **Exclusion:**  Pediatrics, maternity, psychiatric, day surgery | Patients with mixed diseases including CHF  Patients screened by the tool (n=626); HF patients screened by the tool (n=?) | -- |
| Haga et al. 2012  Single center;  Outpatient;  UK (Scotland) | Observational cohort | **Inclusion:**  NYHA class III-IV; Managed by HFNSs team  **Exclusion:**  Patients to be discharged from HF nursing service within 6 weeks | Patients with CHF: LVD, and NYHA III-IV  HF patients screened by the tool (n=138) | **Mean age:**  77 years  **NYHA class:**  III (74%)  IIIb (21%)  IV (5%)  **LVD:**  Mild (19%)  Moderate (36%)  Severe (45%) |
| Gardiner et al. 2013,  Ryan et al. 2013  Multi-center; Hospital inpatient; UK (England) | Cross-sectional | **Inclusion:**  Patients ≥ 18 years; Residents on the ward at 9.00 am on the survey day  **Exclusion:**  Pediatric wards, mother and baby units; Non-English-speaking; Deaf patients; Non-consenting patients; Unable to consent and no one to consent on their behalf | Patients with mixed diseases including CHF  Patients screened by the tool (n=514); HF patients screened by the tool (n=?) | -- |
| Pandini et al. 2016  Single center;  Hospital inpatient; Italy | Observational | **Inclusion:**  Patients admitted to the medical ward from the emergency department | Patients with mixed diseases including HF (probably chronic)  Patients screened by the tool (n=781); HF patients screened by the tool (n=?) | -- |
| RADPAC | | | | |
| Thoonsen et al. 2011,  Thoonsen et al. 2015,  Thoonsen et al. 2019,  Thoonsen et al. 2016  Multi-center; Primary care; Netherlands | Cluster, two-armed randomized controlled trial, followed by a cross-sectional survey (1-year after the trial) and focus group and semi-structured interviews  **Intervention:**  Training GPs on RADPAC and the Problems and Needs Square; coaching sessions with PC consultants; and peer group sessions  **Control:**  Usual patient care | **Inclusion:**  Patients in general practice who died from cancer, CHF,  or COPD in the past 12 months | Patients with mixed diseases including CHF  **Intervention group:**  Patients screened by the tool (n=216); HF patients screened by the tool (n=32)  **Control group:**  Patients screened by the tool (n=0) *(1-year after the trial, patients were screened by the tool by untrained GPs to explore its short-term effect)*  GPs randomized (I=57, C=77); included in the cross-sectional survey (I=12, C=28); included in the interviews (I=9) | -- |
| SPICT | | | | |
| Highet et al. 2014  Single center; Hospital inpatient; UK (Scotland) | Mixed-methods, prospective case-finding | **Inclusion:**  Patients with advanced kidney, liver, heart, or lung disease following an emergency admission to hospital | Patients with mixed diseases including HF  Patients screened by the tool (n=?); HF patients screened by the tool (n=? *“570 patients from the cardiology unit”*) | -- |
| Hamano et al. 2018  Single center; Outpatient; Japan | Cross-sectional | **Inclusion:**  Patients ≥ 65 years | Patients with mixed diseases including HF  Patients screened by the tool (n=87); HF patients screened by the tool (n=? *“5 patients with heart/vascular disease”*) | -- |
| Hamano et al. 2019  Multi-center; Outpatient; Japan | Cross-sectional | **Inclusion:**  Patients ≥ 65 years | Patients with mixed diseases including HF  Patients screened by the tool (n=382); HF patients screened by the tool (n=? *“38 patients with cardiovascular disease”*) | -- |
| NAT:PD-HF | | | | |
| Waller et al. 2013  Single center; Hospital inpatient/  outpatient; Australia | Cross-sectional | **Inclusion:**  HF of systolic or diastolic etiology managed by multidisciplinary HF clinic; Patients receiving maximal therapy or documented intolerance/trial; Hospitalized for HF within the last 12 months; Good English understanding; Cognitive and emotional capacity of participating | Patients with CHF: systolic, diastolic; and NYHA I-IV  HF Patients screened by the tool (n=52) | **Mean age:**  67 years  **NYHA class:**  I (21%)  II (23%)  III (50%)  IV (10%)  **LVEF:**  < 50% (23%)  > 50% (65%) |
| Janssen et al. 2019  Single center; Outpatient; Netherlands | Mixed-methods intervention pilot, including focus group  **Intervention:**  Training on Dutch NAT:PD-HF | **Inclusion:**  Diagnosis of CHF according to the ESC guidelines; NYHA class III-IV; Patients planned to receive a home visit by HFNS; Ability to complete written questionnaires or participate in interviews; Ability to consent  **Exclusion:**  Patients known to the SPC team | Patients with CHF: NYHA III-IV  HF patients screened by the tool (n=23); completed the follow-up outcome measures (n=17)  HFNSs included in the intervention and focus group (n=8) | **Mean age:**  84 years  **NYHA class:**  III (61%)  IV (39%) |
| Campbell et al. 2015,  Campbell et al. 2018  Single center; Hospital inpatient; UK (Scotland) | Observational | **Inclusion:**  Patients ≥ 18 years; Admitted with acute decompensated HF; Diagnosis of HF  **Exclusion:**  Refusal to participate; Inability to consent or complete study assessments (cognitive/language barriers); Readmission; Geographical reasons; Isolated cor pulmonale; Acute coronary syndrome complicated by pulmonary edema | Patients with acute on CHF: HFrEF, HFpEF; valvular heart disease; and NYHA II-IV  HF patients screened by the tool (n=272) | **Mean age:**  75 years  **NYHA class:**  II (30%)  III (52%)  IV (18%)  **LVEF:**  ≤ 50% (67%) |
| NECPAL | | | | |
| Gómez-Batiste et al. 2013,  Gómez-Batiste et al. 2014,  Amblàs-Novellas et al. 2016  Multi-center; Primary care, hospital inpatient, social health center, nursing homes; Spain | Cross-sectional, population-based | **Inclusion:**  Patients with advanced chronic conditions  **Exclusion:**  Outpatient clinics, day-care facilities, and day hospitals *(assuming patients would be identified in primary care centers)* | Patients with mixed diseases including CHF  Patients screened by the tool (n=1,064); HF patients screened by the tool (n=88) | -- |
| de-la-Rica-Escuín et al. 2019  Single center; Hospital inpatient; Spain | Cross-sectional | **Inclusion:**  Patients admitted to internal medicine department; Advanced progressive chronic disease; Charlson score ≥ 3; Patient’s or proxy’s consent | Patients with mixed diseases including CHF  Patients screened by the tool (n=142); HF patients screened by the tool (n=95) | -- |
| Orzechowski et al. 2019  Single center; Hospital inpatient; Brazil | Cross-sectional | **Inclusion:**  Patients > 35 years; Hospitalized in a cardiology service with NYHA class III-IV HF or LVEF ≤ 40%  **Exclusion:**  Unable to respond to tool questions and no one to respond on patient behalf | Patients with HF (probably chronic): HFrEF, and NYHA III-IV  HF patients screened by the tool (n=82) | **Mean age:**  68 years |
| Gastelurrutia et al. 2019  Multi-center; Hospital clinics; Spain | Observational | **Inclusion:**  Ambulatory patients in hospital heart failure clinics | Patients with HF (probably chronic): NYHA I-IV  HF patients screened by the tool (n=922) | **Mean age:**  69 years  **NYHA class:**  I (6%)  II (74%)  III (17%)  IV (3%)  **Mean LVEF:**  41.4% |

C: Control group, CHF: Chronic Heart Failure, COPD: Chronic Obstructive Pulmonary Disease, ESC: European Society of Cardiology, GP: General Practitioner, HF: Heart Failure, HFmrEF: Heart Failure with midrange Ejection Fraction, HFNS: Heart Failure Nurse Specialist, HFpEF: Heart Failure with preserved Ejection Fraction, HFrEF: Heart Failure with reduced Ejection Fraction, I: Intervention group, LVD: Left Ventricular Dysfunction, LVEF: Left Ventricular Ejection Fraction, NYHA: New York Heart Association, PC: Palliative Care, SPC: Specialist Palliative Care

## **Supplemental Table 3** Results of the tools’ applications in identifying heart failure populations who require palliative care and evaluating their needs and participants' perspectives on using the tools

| Author, year | Tools for identifying PC patients and needs | Findings |
| --- | --- | --- |
| IPOS | | |
| Kane et al. 2017,  Kane et al. 2018 | **Patient-identification:**  *NA*  **Needs-identification:**  IPOS *(version-1, patient/7-days version, self-completed by patients with help from HFNSs on request. These nurses were instructed on how to act if patients ask help to fill the tool and supervised by the primary researcher)* | **PC needs in HF patients among those assessed by IPOS (%):**  Anxiety of patients and carers: 12/18 (67%);  Pruritus, insomnia, immobility, leg swelling, cough *(identified by the open question)*: (?)  **Intervention effect:**  Short-term, slight improvement in symptom burden, quality of life, and depression, though not necessarily sustained;  Worsening of caregiver burden  *Statistical significance was not determined because of small sample size*  **Interviews’ findings:**  Strengths: Themes: Identification of unmet needs; Holistic assessment; Patient empowerment  Limitations: No positive effect on clinical interaction; 28% of patients did not consider the intervention to have any clinical effect; HFNSs expressed concern on the IPOS spiritual question *“Have you felt at peace?”* |
| Roch et al. 2020 | **Patient-identification:**  IPOS *(German version, patient/3-days recall, by reporting at least 2 tool items as overwhelming or 3 tool items as severe, self-completed by patients)*  **Needs-identification:**  IPOS *(German version, patient/3-days recall)* | **HF patients identified by IPOS for PC among those screened (%):**  56/100 (56%)  **PC needs in HF patients among those assessed by IPOS (%):**  Clinically relevant (moderate to overwhelming) physical symptoms:  Pain: 40/100 (40%); Shortness of breath (64%); Weakness or lack of energy (62%); Nausea (12%); Vomiting (2%); Poor appetite (40%); Constipation (28%); Sore or dry mouth (68%); Drowsiness (49%); Poor mobility (67%)  Other clinically relevant concerns:  Patient anxiety: 56/100 (56%); Family anxiety (79%); Depression (47%); Feeling at peace (11%); Sharing feelings (42%); Information needs (22%); Practical issues (10%)  **Mean IPOS scores (n=100):**  Total score: 21.5/68 *(insignificant difference between NYHA II and III patients (20.9) versus NYHA IV patients (21.5))*  Physical symptoms score: 13.1/40  Emotional symptoms score: 5.9/16  Communication/practical issues score: 2.5/12 |
| GSF-PIG | | |
| Milnes et al. 2019 | **Patient-identification:**  GSF-PIG *(4^th^ edition, 2011, using only the clinical disease-specific criteria, filled using medical records in hospital wards)*  **Needs-identification:**  *NA* | **HF patients identified by GSF-PIG for PC among those screened (%):**  **~** 10/? (?)  *This represents patients who met the GSF-HF criteria. Number of HF patients who met the other GSF disease-specific criteria, and so were also identified for PC, was not reported. Therefore, the actual number of identified HF patients might be > 10* |
| Haga et al. 2012 | **Patient-identification:**  SHFM, GSF-PIG *(version 2.25, 2006,* *using only the clinical HF-specific criteria, filled by HFNSs who were asked about two of the GSF-HF criteria, and using the clinical HF database for the other two GSF-HF criteria)*  **Needs-identification:**  GSF-PIG *(version 2.25, 2006,* *using only the clinical HF-specific criteria)* | **HF patients identified by GSF-PIG for PC among those screened (%):**  119/138 (86%)  *This represents patients who met the GSF-HF criteria*  **PC needs in HF patients among those assessed by GSF-PIG (%):**  Difficult physical or psychological problems despite optimized therapy: 113/138 (82%);  ≥ 2 hospital admissions for HF symptoms in the previous 12 months: 32/138 (24%);  NYHA class III or IV symptoms: 138/138 (100%)  **Characteristics and outcomes of identified patients (n=119):**  Patients meeting GSF-HF criteria had spent significantly longer in HF service and hospital and had significantly more all-cause hospital admissions over the previous 12 months, compared to those not meeting the criteria. However, they did not have significantly more hospital admissions during the 12-month follow-up period |
| Gardiner et al. 2013,  Ryan et al. 2013 | **Patient-identification:**  GSF-PIG (*using only the clinical disease-specific criteria, filled using hospital case notes by researchers with a clinical background in medicine or nursing after undergoing training in survey methodology and data collection)*  **Needs-identification:**  SPARC | **HF patients identified by GSF-PIG for PC among those screened (%):**  **~** 38/? (?)  *This represents patients who met the GSF-HF criteria. Number of HF patients who met the other GSF disease-specific criteria, and so were also identified for PC, was not reported. Therefore, the actual number of identified HF patients might be > 38*  **Symptom burden in identified patients (n=38):**  61% of identified patients had significant physical burden as measured by SPARC;  39% of identified patients had significant psychological burden as measured by SPARC;  Meeting the GSF-HF criteria was not a predictor of significant physical and psychological burden as measured by SPARC |
| Pandini et al. 2016 | **Patient-identification:**  GSF-PIG *(4^th^ edition, 2011, filled by a physician and nurse of the Internal Medicine ward)*  **Needs-identification:**  *NA* | **HF patients identified by GSF-PIG for PC among those screened (%):**  **~** 20/? (?)  *This represents cardiopulmonary patients who met the tool criteria. However, not all these patients had HF. Therefore, the actual number of identified HF patients might be < 20* |
| RADPAC | | |
| Thoonsen et al. 2011,  Thoonsen et al. 2015,  Thoonsen et al. 2019, Thoonsen et al. 2016 | **Patient-identification:**  RADPAC *(filled by GPs (I) in the trial, and then filled by GPs (C) 1-year after the trial)*  **Needs-identification:**  Problems and Needs Square *(proactive PC planning card)* | **HF patients identified by RADPAC for PC among those screened (%):**  By GPs (I) in the trial: 2/32 (6%)  By GPs (I) 1-year after the trial: 0/? (0%)  By GPs (C) 1-year after the trial, shortly after being administered RADPAC: 4/? (?) *(these GPs did not identify any HF patient before being administered the RADPAC)*  **Intervention effect:**  Number of contacts with out-of-hours GP service, contacts with own GP, hospitalizations, and place of death were not significantly different between patients assessed by GPs (I) and patients assessed by GPs (C)  *This applies to all patients “I=216, C=271”; some of whom had HF “I=32, C=52”*  In post-hoc analysis; patients identified as palliative by GPs (I) had significantly more contacts with their own GP, fewer hospitalizations, and less often died in hospital compared to all other patients. However, number of contacts with out-of-hours GP service was not significantly different  *This applies to all 49 identified patients; 2 of whom had HF*  **Interviews’ findings:**  Strengths: Theme: Identification of palliative patients (clear indicators, integrated into some GPs’ daily practice)  Limitations: Timely recognition of HF patients who need PC was considered difficult despite using RADPAC |
| SPICT | | |
| Highet et al. 2014 | **Patient-identification:**  SPICT *(2013, filled by senior nursing ward staff and specialty registrars)*  **Needs-identification:**  SPICT *(2013)* | **HF patients identified by SPICT for PC among those screened (%):**  **~** 16/570 (3%)  *This represents patients from the cardiology unit who met the tool criteria. However, not all these patients had HF. Therefore, the actual number of identified HF patients might be < 16*  **PC needs in HF patients among those assessed by SPICT (%):**  *Needs were reported for mixed-disease patients, without specifying those for HF patients* |
| Hamano et al. 2018 | **Patient-identification:**  SPICT-J *(Japanese version, 2015, filled by the chief researcher)*  **Needs-identification:**  SPICT-J *(Japanese version, 2015)* | **HF patients identified by SPICT for PC among those screened (%):**  ~ 3/5 (60%)  *This represents* *heart-vascular disease patients who met the tool criteria. However, not all these patients might have HF. Therefore, the actual number of identified HF patients might be < 3*  **PC needs in HF patients among those assessed by SPICT (%):**  Asks for PC or treatment withdrawal: 3/3 (100%);  KPS ≤ 50% and limited reversibility: 1/3 (33%);  Significant weight loss or low body mass index: 1/3 (33%);  Persistent troublesome symptoms: 1/3 (33%);  Care dependence: 0/3 (0%);  Unplanned hospital admissions: 0/3 (0%) |
| Hamano et al. 2019 | **Patient-identification:**  SPICT-J *(Japanese version, 2015, filled by GPs)*  **Needs-identification:**  SPICT-J *(Japanese version, 2015)* | **HF patients identified by SPICT for PC among those screened (%):**  **~** 5/38 (13%)  *This represents cardiovascular disease patients who met the tool criteria. However, not all these patients might have HF. Therefore, the actual number of identified HF patients might be < 5*  **PC needs in HF patients among those assessed by SPICT (%):**  NYHA class III-IV HF, or extensive, untreatable coronary artery disease with breathlessness or chest pain at rest or on minimal exertion: 4/38 (11%) |
| NAT:PD-HF | | |
| Waller et al. 2013 | **Patient-identification:**  *NA*  **Needs-identification:**  NAT:PD-HF *(filled by HF service staff (doctors and nurses) in consultation with patients)* | **PC needs in HF patients among those assessed by NAT:PD-HF (%):**  Physical symptoms: (?) *most reported some concern;*  Daily living activities: (?) *most reported some concern;*  Psychological symptoms: (?) *most reported no concern;*  Social needs: (?) *most reported no concern;*  Spiritual needs: (?) *most reported no concern* |
| Janssen et al. 2019 | **Patient-identification:**  NAT:PD-HF *(Dutch translation, by reporting some/potential or significant concern for any tool item, filled by HFNSs who were collaborating closely with CHF cardiologists (part of a multidisciplinary team) and trained in CHF care and motivational interviewing skills)*  **Needs-identification:**  NAT:PD-HF *(Dutch translation)* | **HF patients identified by NAT:PD-HF for PC among those screened (%):**  23/23 (100%)  **PC needs in HF patients among those assessed by NAT:PD-HF (%):**  *Assessed but proportions were not reported*  **Intervention effect:**  No significant improvement in symptom burden, physical functioning, care dependency, and caregiver burden;  Significant worsening of disease-specific health status;  No effect on number of recorded advance directives, hospital admissions, and emergency room visits  **Focus group findings:**  Strengths: Paying attention to caregiver needs  Limitations: Not helpful to discuss PC needs; Lack of questions that help to introduce and communicate about PC; Questions not fitting with patient needs; Many questions are not clear to patients; Lack of guidance towards required interventions after patient identification; Long list with several questions; Not considering to what extent patients want to discuss PC needs |
| Campbell et al. 2015,  Campbell et al. 2018 | **Patient-identification:**  KCCQ, SF-12, ESAS, HADS, NAT:PD-HF *(by reporting significant concern for any item in tool section-2 “patient wellbeing”, filled by a physician)*  **Needs-identification:**  KCCQ, SF-12, ESAS, HADS, NAT:PD-HF | **HF patients identified by NAT:PD-HF for PC among those screened (%):**  70/272 (26%)  **PC needs in HF patients among those assessed by NAT:PD-HF (%):**  *Assessed but proportions were not reported*  **SPC need in identified patients (n=70):**  38% of patients who met the authors’ definition of “SPC need” were identified by NAT:PD-HF compared to 21% of patients who did not (significant difference);  Being identified by NAT:PD-HF was an insignificant predictor of which patients need SPC according to the authors’ definition  *Authors defined SPC need as “persistently severe impairment of any PROM (KCCQ, SF-12, ESAS, HADS) without improvement, or severe impairment immediately preceding death”* |
| NECPAL | | |
| Gómez-Batiste et al. 2013,  Gómez-Batiste et al. 2014,  Amblàs-Novellas et al. 2016 | **Patient-identification:**  NECPAL *(Catalan version, filled by healthcare professionals (doctors and nurses) for the categories that require clinical judgement, and using clinical records for the quantitative variables)*  **Needs-identification:**  NECPAL *(Catalan version)* | **HF patients identified by NECPAL for PC among those screened (%):**  80/88 (91%)  *65 patients* *met the NECPAL-chronic heart disease criteria (not enough to be identified)*  **PC needs in HF patients among those assessed by NECPAL (%):**  Choice/request for PC by patient: 5/63 (8%);  Choice/request for PC by family: 13/63 (21%);  Need of PC by healthcare professionals: 10/63 (16%);  General clinical indicators: *low level of needs (proportions were reported in detail but no space to display in this table)*  **Other findings:**  98% of heart disease patients with a negative answer to the surprise question were identified for PC by NECPAL |
| de-la-Rica-Escuín et al. 2019 | **Patient-identification:**  NECPAL *(version 3.0, 2016, filled by patients, caregivers, or principal investigators who were trained on using NECPAL (depending on patient condition at that time and the nature of tool items), and using patient’s clinical history)*  **Needs-identification:**  NECPAL *(version 3.0, 2016)* | **HF patients identified by NECPAL for PC among those screened (%):**  > 85/95 (> 89%)  *89 patients* *met the NECPAL-chronic heart disease criteria (not enough to be identified)*  **PC needs in HF patients among those assessed by NECPAL (%):**  *Needs were reported for mixed-disease patients, without specifying those for HF patients*  **Other findings:**  100% of heart disease patients with a negative answer to the surprise question were identified for PC by NECPAL |
| Orzechowski et al. 2019 | **Patient-identification:**  NECPAL *(Catalan version, filled by* *an assistant physician, patient, and/or caregiver)*  **Needs-identification:**  NECPAL *(Catalan version)* | **HF patients identified by NECPAL for PC among those screened (%):**  45/82 (55%)  **PC needs in HF patients among those assessed by NECPAL (%):**  Choice/request for PC by patient/family: 30/82 (37%);  Need of PC by healthcare professionals: 47/82 (57%);  General clinical indicators: *low to moderate level of needs (proportions were reported in detail but no space to display in this table)*;  Specific clinical indicators: *low level of needs (proportions were reported in detail but no space to display in this table)*  **Other findings:**  98% of HF patients with a negative answer to the surprise question were identified for PC by NECPAL |
| Gastelurrutia et al. 2019 | **Patient-identification:**  NECPAL *(Catalan version, filled by* *a nurse and/or physician)*  **Needs-identification:**  NECPAL *(Catalan version)* | **HF patients identified by NECPAL for PC among those screened (%):**  297/922 (32%)  **PC needs in HF patients among those assessed by NECPAL (%):**  Choice/request for PC by patient/family: 4%;  Need of PC by healthcare professionals: 4%;  General clinical indicators: 71% (multimorbidity is the most common);  HF-specific clinical indicators (≥ 2 indicators): 17%   - LVEF < 30% or PAP > 60 mmHg: 19% - NYHA III/IV, severe valve disease, or inoperable coronary disease: 24%   **Other findings:**  91% of HF patients with a negative answer to the surprise question were identified for PC by NECPAL |

C: Control group, CHF: Chronic Heart Failure, ESAS: Edmonton Symptom Assessment System, GP: General Practitioner, HADS: Hospital Anxiety and Depression Scale, HF: Heart Failure, HFNS: Heart Failure Nurse Specialist, I: Intervention group, KCCQ: Kansas City Cardiomyopathy Questionnaire, KPS: Karnofsky Performance Scale, LVEF: Left Ventricular Ejection Fraction, NA: Not Applicable, NYHA: New York Heart Association, PAP: Pulmonary Artery Pressure, PC: Palliative Care, PROM: Patient-Reported Outcome Measure, SF-12: Short Form health survey-12, SHFM: Seattle Heart Failure Model, SPARC: Sheffield Profile for Assessment and Referral to Care, SPC: Specialist Palliative Care
